# Herpes Simplex Virus and Pattern Recognition Receptors: An Arms Race

**DOI:** 10.3389/fimmu.2020.613799

**Published:** 2021-01-29

**Authors:** Jun Zhao, Chao Qin, Yongzhen Liu, Youliang Rao, Pinghui Feng

**Affiliations:** Section of Infection and Immunity, Herman Ostrow School of Dentistry, Norris Comprehensive Cancer Center, University of Southern California, Los Angeles, CA, United States

**Keywords:** herpes simplex virus, pattern recognition receptors, RIG-I/MDA5, CGAS, IFI16, AIM2, DAI, PKR

## Abstract

Herpes simplex viruses (HSVs) are experts in establishing persistent infection in immune-competent humans, in part by successfully evading immune activation through diverse strategies. Upon HSV infection, host deploys pattern recognition receptors (PRRs) to recognize various HSV-associated molecular patterns and mount antiviral innate immune responses. In this review, we describe recent advances in understanding the contributions of cytosolic PRRs to detect HSV and the direct manipulations on these receptors by HSV-encoded viral proteins as countermeasures. The continuous update and summarization of these mechanisms will deepen our understanding on HSV-host interactions in innate immunity for the development of novel antiviral therapies, vaccines and oncolytic viruses.

## Introduction

Herpesviridae is a family of large DNA viruses that establish persistent infection within their immune-competent host. Members of the family are further grouped into three subfamilies, i.e., alpha-, beta-, and gamma-herpesviruses based on their genome organization, biological characteristics, and cell tropism (1, 2). Herpes simplex virus type 1 (HSV-1 or human herpesvirus 1, HHV-1) and type 2 (HSV-2 or human herpesvirus 2, HHV-2) belong to the alpha-herpesvirus subfamily and the genera simplex virus. They are neurotropic viruses that establish latent infection in the trigeminal ganglia (TG) and dorsal root ganglia (DRG) for the entire life of the host (3). Seropositive for HSV are high, averaging nearly 70% in the general population and approaching 100% in senior citizens of 65-year or older (4, 5). Clinical manifestations of HSV-1 infections include various mucocutaneous diseases, such as herpes labialis, genital herpes, herpetic whitlow, and keratitis (6). It can cause encephalitis that is often life-threatening, in a small portion of the infected individuals who are immune-compromised (6). HSV-2 infection frequently causes genital sores (7).

HSV-1 and HSV-2 are structurally closely related. Herpes simplex virions are spherical particles with a diameter of 186 nm, with glycoprotein protrusions on the surface, making the full diameter approximately 225 nm (8). Both viruses contain a linear double-stranded DNA (dsDNA) genome that is ~150 kilobase (kb) in size and encodes more than 70 open reading frames (ORFs). The viral genomes are caged by a 125 nm icosahedral capsid, which is surrounded by an amorphous layer called tegument (9). Packaged within the tegument compartment, a large number of viral structural proteins are released into the infected cell to establish an environment that is conducive for viral replication. The tegument is enveloped by a lipid bilayer within which multiple viral glycoproteins are embedded. These surface glycoproteins mediate the entry and fusion of the virus with the target cell (10).

HSV-1 and HSV-2 share almost identical replication cycles. Viral entry into host cells is mediated by the interactions between cellular receptors and viral glycoproteins anchored within the virion envelope. The initial binding occurs through the binding of envelope glycoprotein C (gC) and/or gB to heparan sulfate proteoglycan, which is immediately followed by gD association with one of the three known receptors to initiate virus entry (11). The receptors involved are cell-type dependent. While nectin-1 is the main receptor of epithelial cells, neuronal cells and fibroblasts (12), HVEM is the main receptor of T cells and cornea epithelial cells, for HSV infection (13, 14). Upon fusion of the virion envelope with the host cell membrane, tegument proteins are released into the cytoplasm of the infected cells to facilitate capsid trafficking and evade host antiviral immunity. The de-enveloped nucleocapsid is transported along microtubules to the nuclear pore, where the viral genome is injected into the nucleus. At this point, HSVs adopt two modes of infection. In neuronal cells located at the peripheral ganglia region and lab-isolated primary neurons, the viral genome stays as a circularized episome with no active gene transcription except for the latent-associated transcripts (LATs) (15). LATs do not encode proteins, but two major RNA species and several small non-coding RNAs that regulate cell survival and viral lytic gene expression (16). Therefore, this stage is termed as viral latency with no clear clinical manifestation. However, the virus can be periodically reactivated and enters the lytic cycle, largely due to stress responses and other stimuli not fully understood. During the lytic cycle, the viral genome serves as the template for transcription, leading to the sequential production of viral messenger RNAs and polypeptides of the immediate early (IE), early (E), and late (L) phases (17). Tegument protein VP16 and cellular factors promote transcription of IE genes [e.g., infected cell polypeptide 0 (ICP0), ICP4, ICP22, ICP27 and ICP47]. IE proteins then promote transcription and translation of E genes, which produce the necessary components for viral DNA replication. Replicated viral genomes collaborate with transcription factors to promote the expression of L proteins that are structural components of HSV virions (such as glycoproteins and capsid proteins VP5, VP21, VP23, VP24 and VP26), thereby maximizing viral protein production in preparation for viral assembly and egress. Nucleocapsids assemble in the nucleus, undergo envelopment and de-envelopment at the nuclear membrane, and re-envelopment in the TGN to acquire their tegument and glycoprotein-embedded membrane, en route to the maturation and release of amplified virion progeny (18). Importantly, viral latency, reactivation and lytic replication collectively contribute to the life-long ‘persistent infection’ of HSV in an immune-competent host, leading to the recurrent pathogenesis associated with the virus.

Upon infection, host cells sense invading viruses *via* cellular pattern-recognition receptors (PRRs) to initiate the antiviral innate immune defense. Structurally, PRRs can be generally classified into several major families, including Toll‐like receptors (TLRs), RIG‐I like receptor (RLRs), NOD‐like receptors (NLRs), C‐type lectin receptors (CLRs), AIM2‐like receptors (ALRs), and cyclic GMP‐AMP synthase (cGAS). These PRRs can recognize various pathogen-associated molecular patterns (PAMPs) from bacteria, viruses, fungi and protozoa. Microbial PAMPs can be lipoproteins, carbohydrates, lipopolysaccharides and nucleic acids. PRRs also recognize endogenous damage- or danger-associated molecular patterns (DAMP) from the host, which are related to immune homeostasis and autoimmune diseases. Among PAMPs, the nucleic acid RNA and DNA have attracted much attention. PRRs recognizing the nucleic acids include: DNA sensors such as endosomal Toll-Like Receptor 9 (TLR9), cytosolic Absent In Melanoma 2 (AIM2), Interferon Gamma Inducible Protein 16 (IFI16), DNA-dependent Activator of Interferon-regulatory factors (DAI) and cyclic GMP-AMP synthase (cGAS); RNA sensors TLR3, TLR7, TLR8, and cytosolic Retinoic acid-Inducible Gene I (RIG‐I), Melanoma Differentiation-Associated protein 5 (MDA5), NLR Family Pyrin Domain Containing 3 (NLRP3), and Nucleotide-binding Oligomerization Domain-containing protein 2 (NOD2) (19). TLRs are transmembrane receptors, while cytosolic or nuclear receptors are soluble within their corresponding compartments. After sensing PAMPs or DAMPs, PRRs activate their adaptors and downstream Interferon Regulatory Factors (IRFs) and Nuclear Factor kappa-light-chain-enhancer of activated B cells (NF-κB), leading to the transcription and translation of cytokines, chemokines, MHC, and co-stimulatory molecules. In addition, PRRs can trigger signal transduction and induce cellular processes that do not rely on transcription, such as phagocytosis, autophagy, cell death, and inflammasome activation. These processes work in concert with innate immune response to mesh a network of antiviral host defense (19). In this review, we will summarize the recent findings on the contribution of cytosolic PRRs to sense HSV in host defense, and the counteractive measures deployed by HSV to deflect these PRRs to establish persistent infection.

## The RIG-I- and MDA5-MAVS Pathway

RLRs, including RIG-I (20), MDA5 (21, 22), and probable ATP-dependent RNA helicase DHX58 (LGP2), are cytoplasmic PRRs that recognize virus-derived or viral infection-associated cellular double-stranded RNA (dsRNA). RIG-I recognizes short, blunt-ended dsRNA carrying terminal 5’-triphosphate or 5’-diphosphate moieties (23), while MDA5 prefers longer dsRNA independent of its terminal phosphate groups.

Upon engaging viral dsRNA, RIG-I and MDA-5 hydrolyze ATP to induce their oligomerization on the dsRNA, thereby exposing their N-terminal caspase activation and recruitment domains (CARDs) to relay immune activation *via* seeding the oligomerization of the adaptor protein MAVS (also known as IPS-1, CARDIF, and VISA) (24–28). LGP2 lacks the CARD domain and is reported to inhibit RIG-I-mediated antiviral responses. Once activated, MAVS forms prion-like oligomers on the outer membrane of mitochondria (29), which further recruits the tank-binding kinase-1(TKB1) and IκB kinase (IKK) complex to activate IRF and NF-κB transcription factors, respectively. Therefore, RIG-I and MDA5 exhibit antiviral activities to a broad spectrum of RNA viruses, including influenza A virus, hepatitis C virus, dengue virus, encephalomyocarditis virus, coronavirus, etc (30). Post-translational modifications, such as phosphorylation and ubiquitination, are discovered to tightly regulate the activation of RIG-I (31–35).

Unlike RNA viruses, genomes of DNA viruses such as herpes simplex viruses (HSV-1 and -2) do not carry the structural features required for binding to RLRs. Remarkably, RLRs demonstrate antiviral activities against HSVs. During HSV-1 latency, two small non-coding RNAs (sncRNAs) coded by the LAT, sncRNA1 and sncRNA2, were shown to interact with and activate RIG-I in neuronal cells, resulting in type I interferon induction and NF-κB activation that promote viral latency and neuronal survival (36). Upon viral entry, early studies have shown that RIG-I and MDA5 non-redundantly activate type I IFN genes upon cytosolic DNA stimulation (37). In support of this, DNA-dependent RNA polymerase III (Pol III) is reported to convert cytosolic DNA to 5’-ppp RNA that activates RIG-I (38). Regarding the source of cytosolic DNA, in macrophages, HSV-1 capsid is found to be degraded by the ubiquitin-mediated proteasome system, thereby releasing viral DNA into the cytosol (39). As such, RIG-I and TLR9 is reported can cooperate to enable the production of type I IFN in HSV-2–infected mouse macrophages (40). However, MDA5 mediates a Pol III-independent pathway to sense HSVs in primary human macrophages (41). The identity of viral RNA or other ligands activating MDA5 remains unknown. In nonimmune cells infected with HSV, studies have detected dsRNA localized in the cytosol, which activates the RIG-I-mediated IFN induction (42). It is proposed that dsRNA molecules originated from the complementary transcription of HSV activate RIG-I. Interestingly, transcripts derived from a cellular 5S ribosomal RNA pseudogene are found to be unmasked by HSV-1 to induce RIG-I activation (43). These findings collectively support the role of RIG-I and MDA5 to sense herpes simplex viruses and induce IFN response.

To counteract RIG-I- and MDA-mediated type I IFN responses, HSV has evolved strategies to directly target these receptors. HSV-2 virion host shutoff (Vhs) protein selectively suppresses the expression of TLR2, TLR3, RIG-I and MDA-5 in human vaginal epithelial cells (44). Given that Vhs is not a sequence-specific endonuclease, it remains unknown how Vhs selectively targets these mRNAs of innate immune function for destruction. It was shown that Vhs targets mRNA for degradation, *via* associating with translation initiation factors (45, 46). Thus, infection-induced translational activation of mRNAs of immune function may be preferentially degraded by Vhs. US11, a dsRNA-binding protein packaged in the virion, binds to RIG-I and MDA5 in a manner independent of its RNA-binding domain and inhibits their interactions with MAVS (47). Released from the tegument upon infection, UL37 displays an intrinsic enzyme activity to deamidate RIG-I during HSV-1 infection (42). Deamidation of two asparagine residues in the helicase domain of RIG-I abrogates its binding to dsRNA and subsequent RNA-stimulated helicase activity. As such, recombinant HSV-1 containing a point mutation that abolishes UL37 deamidase activity triggers more robust RIG-I activation and potent IFN responses than wild-type HSV-1. This recombinant HSV-1 is highly attenuated *in vitro* and in mice.

## The cGAS-STING-IFN Pathway

Stimulator of interferon genes (STING), also known as Met-Pro-Tyr-Ser (MPYS), mediator of IRF3 activation (MITA) (48), Endoplasmic Reticulum IFN stimulator (ERIS) (49), transmembrane protein 173 (TMEM173), is an endoplasmic reticulum adaptor that mediates innate immune activation in response to cyclic dinucleotides (CDNs) (48, 50). These CDNs include cyclic-di-AMP, cyclic-di-GMP, and cyclic-GMP-AMP. Upon activation, STING oligomerizes and translocates to the trans-Golgi network (TGN) where STING recruits TBK1 and IKK kinase complex to activate IRF and NF-κB, leading to the production of type I interferons and inflammatory cytokines. Notably, K27- and K63-linked polyubiquitin chains of STING are essential for the activation of the transcription activity of IRF3 (51).

In response to HSV-1 infection, STING is required for IFN production in multiple cell lines, including murine embryonic fibroblasts, macrophages and dendritic cells (52). Moreover, STING protects mice from HSV-1 lethal infection *via* intravenous and intracerebral routes, while mucosal infection of HSV-1 in *STING* ^−/−^ mice results in the increased corneal and trigeminal ganglia viral titers, demonstrating the importance of STING in host defense against HSV-1 *in vivo* (53). As one of the countermeasures, HSV-1 deploys UL36 (also known as VP1–2) to deubiquitinate STING, thus impeding the activation of TBK1 and IRF3. UL36 is the largest protein encoded within HSV and likely provides a scaffold for tegument protein incorporation (54). In fact, HSV-1 ΔDUB mutant induces more robust IFN induction in microglia and shows reduced replication in the brain compared with wild-type HSV-1 (55). Besides UL36, _γ1_34.5 (ICP34.5) interacts with STING and disrupts its translocation from endoplasmic reticulum to Golgi apparatus, a step that is essential for STING to transduce innate immune signals (56). Lastly, ICP27, expressed during HSV-1 *de novo* infection in macrophages, interacts with the activated TBK1-STING signalosome to inhibit IRF3 activation (57), thereby evading immune response downstream of STING.

Paradoxically, in several cell lines, including HEp-2 and HeLa, STING is found to be stabilized by HSV-1 viral proteins, and depletion of STING impedes HSV-1 productive infection (58). The mechanism by which STING enhances HSV-1 replication in these cell lines remains unclear. Nevertheless, these findings suggest the opposing function of STING in host defense is cell type-dependent. One possibility is that the STING-dependent immune defense pathway is rewired by the tumor cell to promote proliferation or growth, which is usurped by HSV-1.

Cyclic guanosine monophosphate (GMP)-adenosine monophosphate (AMP) synthase (cGAS), is a sensor that binds to virus or cell-associated DNA in a sequence-independent manner (59). cGAS is previously demonstrated to mainly reside in the cytoplasm to detect cytoplasmic DNA as it represents a danger signal. Recent finding also suggests that cGAS enters the nucleus to inhibit DNA double-stranded breaks and promotes tumorigenesis (60). The binding of cGAS to DNA induces its oligomerization and concomitant conformational changes, enabling its enzymatic domain to catalyze the synthesis of a second messenger, cyclic GMP-AMP (cGAMP), from cellular GTP and ATP. cGAMP serves as a ligand to activate STING and the downstream IRF and NF-κB branched pathways (61). Therefore, the cGAS-STING pathway plays pivotal roles in inducing type I IFNs and cytokines to mount innate immune responses against bacterial, DNA viruses, cellular genome instability and other related danger signals.

Soon after its discovery, the contribution of cGAS to antagonize HSV-1 was demonstrated by that cGAS^−/−^ mice were more susceptible to HSV-1 challenge than wild-type mice (62). cGAS deficiency also led to impaired IFN expression in microglia, thus resulting in the susceptibility of the mice to herpes simplex encephalitis (HSE) upon ocular infection (63). As cGAS senses HSV-1 DNA to trigger innate immune responses, it is not surprising that HSV-1 evolved diverse strategies to antagonize this pattern recognition receptor and its downstream signaling. HSV-1 tegument protein UL41, an mRNA-specific endonuclease, downregulates the mRNA and protein level of cGAS to abrogate cGAS- and STING-mediated signaling (64). In addition, another tegument protein VP22 is found to interact with cGAS and directly inhibit its enzymatic activity (65). β-catenin is found to be required for the optimal induction of IFN induced by cGAS. As such, HSV-1 US3 phosphorylates β-catenin at Thr556 and blocks its nuclear translocation to dampen cGAS-dependent host antiviral responses (66). We identify that HSV-1 tegument deamidase UL37 targets cGAS, in addition to RIG-I, for deamidation (67). Deamidation of N210, which is in close proximity to the catalytic triad of cGAS, abolishes its catalytic activity to synthesize cGAMP, thereby shutting down cGAMP production and downstream signaling. Interestingly, deamidation does not impair DNA-binding and oligomerization of cGAS, implying the dominant negative effect of deamidated cGAS on the cGAS-IFN pathway. Importantly, non-human primates are resistant to HSV-1 infection and their cGAS proteins contain histine or arginine at the equivalent location of residue 210, which makes cGAS resistant to HSV-1–induced deamidation (67). These findings suggest that cGAS deamidation contributes to the host susceptibility of HSV-1. Altogether, our studies highlight the utmost immune evasion functions of UL37 by targeting multiple sensors for deamidation.

## IFI16

IFI16 belongs to the IFN-inducible PYHIN-200 gene family. Members in this family carry the signature HIN domain (IFI16 has two) that binds to dsDNA or ssDNA in a sequence-independent manner. In addition to a DNA-binding domain, IFI16 contains a PYRIN domain (PYD) that mediates protein-protein interactions. Binding to DNA can trigger two distinct signaling pathways, i.e., IFN signaling and inflammasome signaling, depending on the nature of the stimulating signal (68). During viral infection, IFI16 is proposed to bind viral DNA and trigger the activation of STING and induction of IFN, although the detailed mechanism remains unknown (68).

Depletion of IFI16 in the cornea by *in vivo* siRNA transfection results in the decrease of IRF3 phosphorylation and correspondingly increase of HSV-1 viral replication, while MyD88^−/−^ and Trif^−/−^ double knockout mice demonstrate similar IFN production compared to WT controls. This result suggests that IFI16, rather than TLRs, mediates the innate immune response in corneal epithelium against HSV-1 (69). Unlike the cornea, IFI16 is largely dispensable for host defense against HSV-2 in the urogenital system, while TLR2, TLR9, and DAI are essential for IFN and cytokine production (70). In primary human foreskin fibroblasts (HFFs), nuclear resident IFI16 senses the HSV-1 DNA to induce IFN production in a STING-dependent manner, while cGAS promotes IFI16-mediated IFN induction *via* stabilizing IFI16 protein (71). However, how nuclear IFI16 triggers STING activation remains to be addressed. Another study reports a different mechanism of IFI16 to restrict HSV-1 replication in multiple cell lines, where IFI16 selectively binds to HSV-1 transcription start sties to block viral gene transcription *via* inducing repressive histone modifications (72). These studies demonstrate multiple functions of IFI16 to restrict HSV-1 replication. The controversy on IFI16 and cGAS as the HSV-1 sensor could be explained by the differential compartmentalization of the two sensors. For example, cGAS plays major roles in sensing HSV-1 DNA in macrophages, where viral DNA is exposed in the cytosol due to capsid degradation (39). In contrast, IFI16 may detect and mount innate immune response in cells where DNA is delivered into the nucleus. However, a number of recent studies reported that part of the cGAS resides in the nucleus (60, 73), adding to the complexity of the nuclear DNA-sensing mechanism against HSV-1. Following the sensing of nuclear DNA, an immediate question is how nuclear signal of activated IFI16 is relayed to the cytoplasmic STING and downstream signaling events. These questions call for further investigation.

To counteract IFI16, HSV-1 encodes ICP0, a E3 ligase, to induce IFI16 degradation in a proteasome-dependent manner (74). Interesting, ICP0 reduces IFI16 protein in HFFs and oral keratinocytes (NOKs), whereas HSV-1–induced loss in IFI16 protein is dependent on Vhs-mediated mRNA decay (75). These findings highlight distinct mechanisms by which HSV-1 antagonizes the expression of IFI16 in a cell type-specific fashion.

## AIM2

Transfection of bacterial, viral and cellular DNA into macrophages leads to the formation of inflammasomes (76, 77). The inflammasome is a protein complex formed in response to the activation of several sensors, including the NLR (NOD-like receptor) or PYHIN (containing pyran and HIN domains) proteins upon recognizing varieties of viral PAMPs (78). Genetic manipulation *via* RNA interference in cultured cells and knockout in mice demonstrates that AIM2 is a cytosolic DNA sensor (79–84). AIM2 consists of a C-terminal HIN-200 domain and an N-terminal pyrin domain, which form an intramolecular loop to establish a self-repressing state (85). Upon stimulation, the HIN-200 domain binds directly to the sugar-phosphate backbone of dsDNA, releasing the pyrin domain which forms homotypic interaction with the pyrin domain of apoptosis-associated speck-like protein containing a carboxy-terminal CARD (ASC) (86). The CARD of ASC then interacts with the CARD of pro-caspase-1 to activate caspase-1 and form the AIM2 inflammasome. Finally, the activated caspase-1 cleaves the pro-IL-1β and pro-IL-18 and induces the release of the mature IL-1β and IL-18 from the cell (85, 87). Importantly, the expression of the sensors and the cytokine precursors requires a priming step that is stimulated by pro-inflammatory signals such as LPS. Besides cytokine releasing, activated AIM2 inflammasome also induces an inflammatory cell death to protect infected host from invading pathogens, including intracellular bacteria (88, 89), vaccinia virus (79, 89), and murine cytomegalovirus (a beta-herpesvirus) (89). In the absence of microbial infection, AIM2 also plays an important role in sensing damage-associated molecular patterns (DAMPs) released by distressed or damaged cells (90). The cellular DNA, as one of the DAMPs produced by nuclear DNA damage or immunogenic cell death, activates AIM2 and initiates inflammasome assembly to promote the secretion of IL-1β and IL-18 (91–93). It was reported that inhibition of potassium efflux inhibited the secretion of IL-1β mediated by AIM2 (94), suggesting that like NLRP3, AIM2 inflammasome activation may depend on distinct ion fluxes and concentrations (95).

Because viral DNA can be released into the cytoplasm during HSV-1 infection in macrophages, it should engage cytoplasmic DNA sensors such as AIM2 (39, 96). However, HSV-1 infection of macrophages induces inflammasome activation independent of AIM2, in stark contrast to murine cytomegalovirus that efficiently induces AIM2-dependent inflammasome activation (89). Based on this observation, it is hypothesized that HSV-1 may have evolved a mechanism(s) to evade AIM2-dependent inflammasome activation. Indeed, HSV-1 tegument protein VP22 was reported to inhibit AIM2-dependent inflammasome activation and IL-1β secretion in infected macrophages (97). VP22 interacts with AIM2 and prevents its oligomerization, an essential step in AIM2 inflammasome activation. Consequently, recombinant VP22-deficient HSV-1 (HSV-1ΔVP22) potently induces AIM2 inflammasome activation and subsequent secretion of IL-1β and IL-18. Similarly, HSV-2 and PRV VP22 homologues also demonstrate inhibitory effect on AIM2-dependent inflammasome activation (97). Interestingly, KSHV tegument protein ORF63 interacts with an inflammasome sensor NLRP1 and prevents its oligomerization to block inflammasome activation in ways similar to VP22 (98). Collectively, these findings reveal a mechanism that the inhibition of AIM2-dependent inflammasome activation appears to be shared by diverse herpesviruses.

## DAI

DNA-dependent activator of IRFs (DAI, also known as ZBP-1) is the first putative cytosolic DNA receptor identified (99). DAI recruits TBK1 and IRF3, and induces type I IFN production after binding to dsDNA. HSV-1 induces DAI activation in the murine fibroblast cell line L929 (99). Structurally, DAI contains tandem amino-terminal Z-DNA-binding domains, Zα1 and Zα2 (also called Zβ), which binds double-stranded Z-form DNA (99, 100). In addition to Z-DNA-binding domains, DAI also contains RIP homotypic interaction motifs (RHIMs) that trigger necroptosis and activate NF-κB pathway by interacting with the receptor-interacting kinase-3 (RIPK3) (101, 102). RIPK3 and its downstream substrate Mixed Lineage Kinase domain-Like protein (MLKL) contributes to the programmed necrotic cell death, which curtails viral replication and restricts dissemination of virions (103, 104). In this pathway, DAI acts as a nucleic acid sensor to detect viral RNA transcripts rather than the cytoplasmic viral DNA during the infection of influenza (105–108), vaccinia (109), MCMV (110, 111), and HSV1 (112, 113), and triggers necroptosis. On the other hand, these viruses manage to inhibit necroptosis by encoding gene products to target DAI-mediated signaling (109, 114, 115). MCMV M45 inhibits virus-induced necroptosis by blocking DAI-dependent oligomerization and activation of RIPK3 (115), while HSV-1 deploys ICP6 (UL39) to prevent the formation of DAI-RIPK3-MLKL complex induced by virus infection (116). Therefore, MCMV and HSV1 deploy similar strategies to block DAI-mediated necroptosis and maintain the viability of the infected cells.

## PKR

DsRNA-dependent protein kinase R (PKR), an interferon-stimulated serine/threonine kinase, is a potent antiviral protein whose activity depends on dsRNA binding (117, 118). PKR consists of two dsRNA-binding domains (dsRBDs) and a kinase domain (119). Encountering dsRNA, DsRBDs bind to the backbone of a RNA in a sequence-independent manner, thus triggering a conformational change and subsequent oligomerization of PKR (120). PKR undergoes cis-phosphorylation within the activation loop by its kinase domain (121). Once activated, PKR phosphorylates the translation initiation factor eIF2α, leading to the suppression of eIF2 in cap-dependent translation and a global shutdown of translation (122). As such, PKR exerts its antiviral activity on a broad spectrum of DNA and RNA viruses by blocking the translation of cellular and viral mRNAs. Besides inhibiting protein synthesis, PKR was reported to promote the RLR-mediated type I interferon signaling *via* phosphorylation of IκB (123) and stabilization of mRNAs of type I interferon genes (124). Nevertheless, the molecular mechanism underpinning the PKR-dependent amplification of interferon signaling is not fully understood.

In HSV-1–infected cells, PKR is shown to be activated, which is required for the activation of NF-κB (125). It remains unclear whether dsRNAs activating PKR originate from HSV-1–encoded symmetrical transcripts or the HSV-1–infected host genome. Interestingly, HSV-1 may activate PKR *via* a cellular protein activator known as PACT (126). To escape PKR-mediated antiviral responses, HSV-1 deploys _γ1_34.5 (ICP34.5) to recruit cellular protein phosphatase 1α (PP1) that counteracts PKR-mediated eIF2α phosphorylation and restores translation (127–129). Moreover, ICP34.5 antagonizes Beclin 1-mediated autophagy, an antiviral process that is dependent on PKR (130). US11, a tegument protein, directly binds PKR to inhibit its conformational change and activation by PACT (126). It was later demonstrated that virion-associated US11, rather than its expression during replication, mediates the inhibition of PKR autophosphorylation (131). Additionally, Vhs degrades RNAs to block PKR activation during early stages of HSV-1 infection (132). These diverse viral strategies to antagonize PKR further emphasize the importance of PKR as a potent anti-HSV molecule.

## Discussion

**Figure 1:**
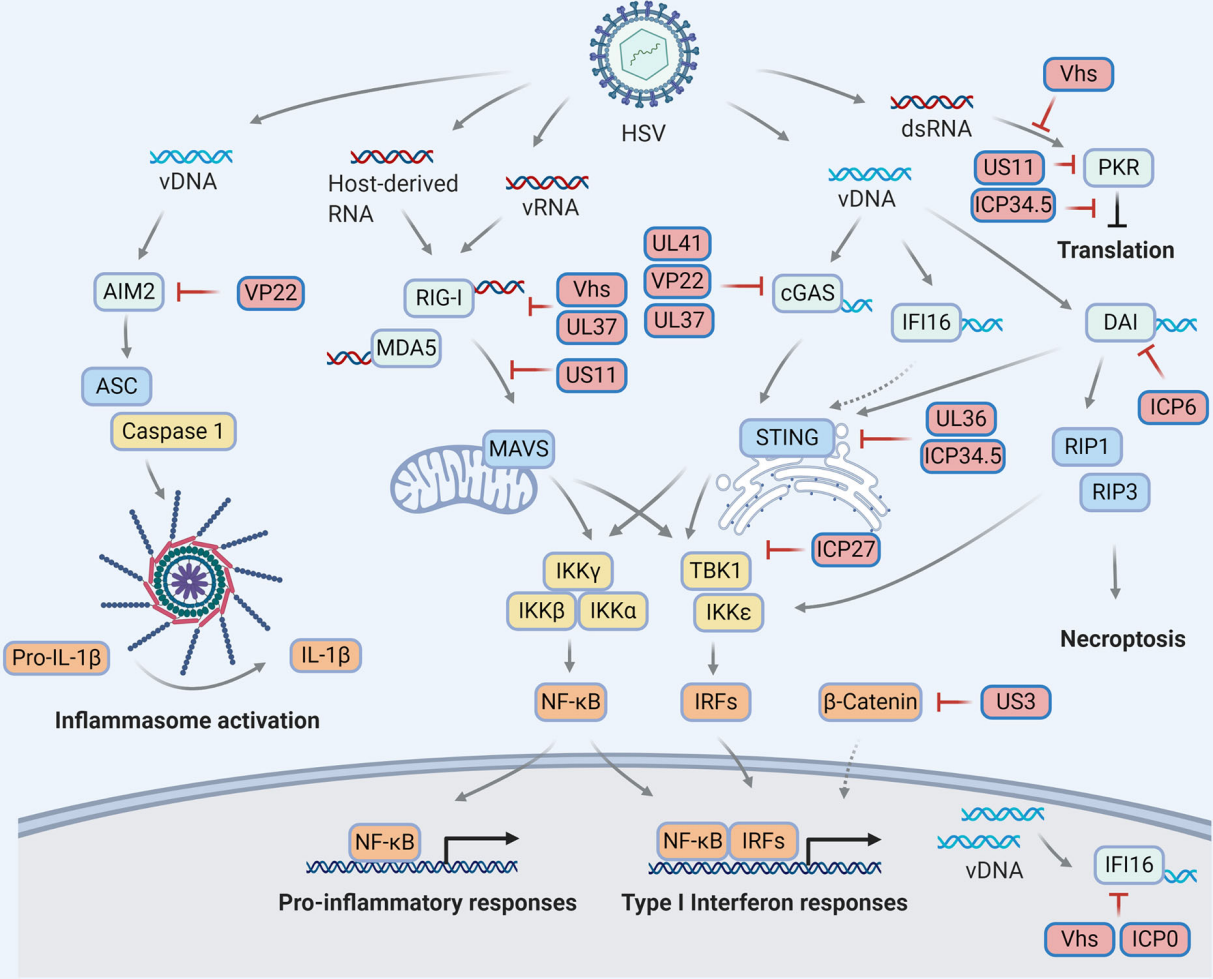
HSV manipulations on the cytosolic pattern recognition receptors. Viral infection derives molecular patterns (DNAs and RNAs) which activate pattern recognition receptors (light blue) to transduce innate immune signaling through distinct adaptor proteins (blue) and ultimately trigger antiviral responses, including but not limited to cytokine production, inflammasome activation, translational inhibition and necroptosis. To escape innate immune surveillance, HSV encode viral proteins (red) to manipulate multiple steps of each signaling pathway *via* diverse mechanisms, resulting in a complex HSV-host interaction network on innate immunity.

In the current review, we summarized the recent findings on the contribution of cytosolic PRRs in sensing HSVs and the direct countermeasures evolved by these viruses (**Figure 1**). During HSV-1 infection, diverse molecular patterns throughout the virus life cycle, including viral DNA genome, transcription-derived RNA species, unmasked cellular RNA, etc., are dynamically sensed by the PRRs to trigger innate immune signaling. On the other side of the coin, HSV develops various countermeasures, ranging from transcription shutoff, protein degradation, interaction competition to enzymatic activity disruption, to escape PRR detection. These lessons learnt from our characterization of HSV-PRR interactions deepen the understanding of the nature and regulations of PRR-mediated innate immune signaling, and may lead to the discovery of novel antiviral modalities. Importantly, strategies interfering with these manipulations can be potentially developed into novel antiviral therapies, while immune modulatory-deficient HSV mutants are good candidates for vaccine and oncolytic virus strains, further highlighting the translational value of the basic research.

One of the knowledge gaps to fill is on the functional redundancy of the PRRs in sensing HSV, as controversy remains on defining the ‘true’ sensor for HSV. While a simple explanation is that such redundancy may have been evolved by the host as backup protections during the arms races with the virus, emerging studies have implicated these PRRs have unique roles in mounting immune responses and antagonizing HSV-1 infection in a temporal and cell/tissue-specific manner. Notably, part of the previous studies relies heavily on a single model cell line, sometimes cancer cell lines, to characterize HSV-PRR interactions, which limits the scope of the findings as some PRRs or signaling pathways may be missing. Thus, more investigations are needed to systematically address the contributions of PRRs, including more *in vivo* studies of HSV infection using tissue-specific knockout mouse models.

Interestingly, ‘functional redundancy’ applies to the virus too, because HSVs deploy multiple proteins to target the same sensor, e.g. RIG-I and cGAS, though *via* distinct molecular mechanism. One possibility is that these viral proteins sequentially work on the sensor throughout the HSV life cycle to maintain constant immune evasion. Alternatively, these viral proteins are cooperating to synergistically antagonize PRR functions or operate in a tissue-specific manner. It will require more work to define the ‘major players’ in these viral proteins that potently antagonize innate immune responses, as efforts in manipulating such proteins confer the greatest susceptibility of the virus to immune response and thus could serve as the best antiviral strategy.

## Author Contributions

JZ and PF conceived the paper. JZ, CQ, YL, YR, and PF wrote the paper. All authors contributed to the article and approved the submitted version.

## Funding

Work in the Feng laboratory is supported by grants from National Institute of Health (DE027556, DE026003 and CA221521 to PF and DE028973 to JZ) and startup funds from the Herman Ostrow School of Dentistry of University of Southern California.

## Conflict of Interest

The authors declare that the research was conducted in the absence of any commercial or financial relationships that could be construed as a potential conflict of interest.
